# The effect of severe and moderate hypoxia on exercise at a fixed level of perceived exertion

**DOI:** 10.1007/s00421-019-04111-y

**Published:** 2019-03-01

**Authors:** Owen Jeffries, Stephen David Patterson, Mark Waldron

**Affiliations:** 1 Faculty of Medical Sciences, School of Biomedical Sciences, Newcastle University, Newcastle Upon Tyne, NE2 4HH UK; 2 School of Sport, Health and Applied Science, St Mary’s University, Twickenham, London, TW1 4SX UK; 3 School of Science and Technology, University of New England, Armidale, NSW Australia

**Keywords:** Arterial oxygen saturation, Hypoxemia, Cognition, Ventilation, Exercise, Altitude

## Abstract

**Purpose:**

The purpose of this study was to determine the primary cues regulating perceived effort and exercise performance using a fixed-RPE protocol in severe and moderate hypoxia.

**Methods:**

Eight male participants (26 ± 6 years, 76.3 ± 8.6 kg, 178.5 ± 3.6 cm, 51.4 ± 8.0 mL kg^− 1^ min^− 1^$\dot {V}$O_2max_) completed three exercise trials in environmental conditions of severe hypoxia (F_I_O_2_ 0.114), moderate hypoxia (F_I_O_2_ 0.152), and normoxia (F_I_O_2_ 0.202). They were instructed to continually adjust their power output to maintain a perceived effort (RPE) of 16, exercising until power output declined to 80% of the peak 30-s power output achieved.

**Results:**

Exercise time was reduced (severe hypoxia 428 ± 210 s; moderate hypoxia 1044 ± 384 s; normoxia 1550 ± 590 s) according to a reduction in F_I_O_2_ (*P* < 0.05). The rate of oxygen desaturation during the first 3 min of exercise was accelerated in severe hypoxia (− 5.3 ± 2.8% min^− 1^) relative to moderate hypoxia (− 2.5 ± 1.0% min^− 1^) and normoxia (− 0.7 ± 0.3% min^− 1^). Muscle tissue oxygenation did not differ between conditions (*P* > 0.05). Minute ventilation increased at a faster rate according to a decrease in F_I_O_2_ (severe hypoxia 27.6 ± 6.6; moderate hypoxia 21.8 ± 3.9; normoxia 17.3 ± 3.9 L min^− 1^). Moderate-to-strong correlations were identified between breathing frequency (*r* = − 0.718, *P* < 0.001), blood oxygen saturation (*r* = 0.611, *P* = 0.002), and exercise performance.

**Conclusions:**

The primary cues for determining perceived effort relate to progressive arterial hypoxemia and increases in ventilation.

## Introduction

Exercise performance during an acute exposure to hypoxia is impaired via a reduction in arterial oxygen content (Fulco et al. 1996, 1998; Calbet et al. 2003a; Amann et al. 2006b; Romer et al. 2006). In moderate hypoxia, where the oxygen fraction of inspired air is reduced to ~ 13–15% (F_I_O_2_ 0.13–0.15), decrements in performance have been attributed to a rise in peripheral markers of muscle fatigue, which generate afferent feedback to down-regulate motor output from the central nervous system (CNS) (Amann et al. 2006b, 2007; Romer et al. 2007). A so-called ‘sensory limit’ (Gandevia 2001), therefore, restricts the manifestation of peripheral fatigue to prevent catastrophic failure of any one system. In severe hypoxia (F_I_O_2_ < 0.115), larger reductions in exercise capacity have been described despite relatively less evidence of peripheral fatigue (Amann et al. 2007). Here, a hypoxia-sensitive ‘central’ component of fatigue mediates a reduction in central motor output via brain hypoxia (Subudhi et al. 2009; Vogiatzis et al. 2011; Millet et al. 2012; Goodall et al. 2012), thus limiting maximal exercise capacity. Indeed, in experiments where F_I_O_2_ is increased at the point of task failure, exercise performance can be prolonged in severe and moderate hypoxia (Amann et al. 2007; Torres-Peralta et al. 2016).

Central processing of the perception of effort and its role in setting exercise intensity is heavily debated. The subjective rating of perceived exertion, termed RPE, is a psychophysiological concept (Borg 1982; Morgan 1994) that centrally integrates perceptual, peripheral, experiential, and environmental sensory cues (Hampson et al. 2001). Indeed, Borg (1982) described the RPE as a conscious representation of multiple inputs. To further understand how perceived exertion modulates self-regulated exercise, a fixed-RPE protocol was developed, referred to as the RPE clamp (Tucker et al. 2006). Here, participants exercise at a pre-determined fixed level of perceived exertion on the RPE scale (typically 16, < ‘very hard’) and modulate their workload according to the perceived mismatches between the expected and actual RPE (Tucker 2009). In an eloquent design, a recent study controlled the rate of arterial hypoxemia (SpO_2_ 98 to 70%) through manipulations in F_I_O_2_, demonstrating that the rate of decline in power output was reliant on the rate change of arterial oxygenation during self-regulated exercise performance (Farra et al. 2017). Whilst this provides evidence of the relationship between SpO_2_ and the perception of exercise intensity in contrived ambient conditions, it overlooks the responses under fixed reductions in F_I_O_2_, such as that commonly encountered at altitude. This is important, since exposure to steady-state F_I_O_2_ conditions provides an opportunity for physiological compensations, such as increased muscle oxygen delivery or extraction. Based on their findings, the rate of change in SpO_2_ under steady-state F_I_O_2_ is likely to determine exercise perception and tolerance, yet this is not currently known. These collective organ-level changes could feasibly offset the deleterious effects of hypoxia or, more importantly, complicate the afferent feedback process. We hypothesized that, during exercise at a fixed RPE, power output would decrease in accordance with a reduction in F_I_O_2_. Therefore, we examined exercise performance using a fixed RPE protocol in severe and moderate hypoxia relative to normoxia, with the aim of determining the relationship between time to exhaustion and a combination of acute physiological responses.

## Materials and methods

### Participants

Eight male participants volunteered to take part in this study (mean ± SD: age 26 ± 6 years; body mass 76.3 ± 8.6 kg; stature 178.5 ± 3.6 cm; maximal oxygen consumption, $\dot {V}$O_2max_ 51.4 ± 8.0 mL kg^− 1^ min^− 1^). All participants were sea-level residents and none had recently travelled to altitude in the 3 months prior to the study. Written informed consent was obtained from each participant. Participants were instructed to avoid consumption of alcohol or caffeinated products for 24 h before each visit, as well as strenuous exercise 48 h before testing and to arrive fully hydrated. All participants gave written informed consent. Ethical approval was provided by the St Mary’s University ethics committee (ref: SMEC_2017-18_012), which was conducted in accordance with the 1964 Helsinki declaration.

### Study design

A randomized, single-blind, crossover design was adopted to examine the effect of breathing different oxygen fractions of air (F_I_O_2_) on exercise at a fixed level of perceived exertion using an RPE-clamp protocol (Tucker et al. 2006). *A-priori* sample size was calculated using G*Power (Version 3.1.9.3). This was determined according to cycling performance in moderate hypoxia (F_I_O_2_ = 0.13) relative to normoxia (F_I_O_2_ = 0.20) at ~ 80% maximum work rate (mean difference 4.5; pooled SD 2.24) (Goodall et al. 2012). Eight participants per group were deemed sufficient to yield a power of 0.82 at *α* = 0.05. The three environmental conditions were randomized by block randomization for groups of three participants at a time using online software (Urbaniak and Plous 2015). Participants visited the laboratory on four separate occasions, each separated by 1 week. During visit 1, participants conducted baseline testing to establish $\dot {V}$O_2max_ and to familiarise with exercising at an RPE of 16 on the 15-grade Borg scale (Borg 1982) in ambient air. During visits 2–4, participants completed the RPE-clamp protocol in variable F_I_O_2_ (severe hypoxia, F_I_O_2_ = 0.114; moderate hypoxia, F_I_O_2_ = 0.152; ambient air, F_I_O_2_ = 0.202).

## Experimental procedures

### Preliminary testing and familiarisation

All cycling exercise was performed on an electronically braked cycle ergometer (Excalibur Sport, Lode, Groningen, The Netherlands). The cycling setup was recorded on the first visit and replicated for subsequent visits. Participants performed a 5-min warm-up at 100 W and a fixed cadence of 80 r min^− 1^. The incremental ramp test began at 120 W and workload increased at a rate of 24 W min^− 1^ until volitional fatigue. Expired gases were collected and highest average 30 s reported as $\dot {V}$O_2max_. RPE was measured at the end of each 1-min stage by pointing to a 6–20 RPE scale, which was held by an investigator. Following the incremental ramp test, two familiarisation exercises were conducted, which were used with the intention of calibrating the participant’s RPE-based selection of power output in the main trials. The first exercise was replicated before every test and began at 120 W, with participants controlling resistance on the ergometer, whilst being blinded to actual power output. The aim was to achieve an RPE that they perceived as equalling RPE-16 over a period of 3–5 min. The test was stopped when participants indicated that they had reached the desired work intensity. The second test began at 20% below the power output selected in test 1 and participants were asked to regulate resistance on the ergometer, by manually toggling up or down on the ergometer’s controls, to maintain an RPE of 16. Participants were also given significant time to discuss and understand the RPE protocol with the researchers both before and after these initial familiarisation trials.

### Fixed-RPE protocol

Participants performed three randomized experimental trials, separated by 1 week in a hypoxic chamber (Sporting Edge, Basingstoke, UK). For each participant, the experimental trials were conducted at the same time of day to eliminate the effect of circadian variation. Participants performed a 5-min warm-up at 100 W and a fixed cadence of 80 r min^− 1^. During experimental testing, the cycling ergometer was in hyperbolic mode, whereby the participant could adjust their power output. No visual feedback was provided, except for cadence. After being fitted with a near-infrared spectroscopy (NIRS) optode, a heart rate chest strap, and facemask, the participants entered the chamber. Participants immediately completed the standardised RPE-ramp protocol to establish setting of RPE over a 3–5-min period. Participants then rested for 5 min to collect baseline data. Finally, they began the fixed-RPE protocol, freely regulating their power output, starting 20% below their self-selected RPE, as determined in the standard ambient air during their first visit. Participants were instructed to cycle at a power output that was perceived to represent an RPE of 16 on the 15-grade Borg scale (Borg 1982) and to adjust their power output, such that an RPE of 16 was maintained. An RPE of 16 represents a verbal cue of between ‘hard’ and ‘very hard’ on the Borg Scale. During the fixed-RPE trial, the highest power output achieved during a 30-s period in each condition was recorded and participants exercised until their power output declined to 80% of this initial value. The trial was stopped when power output fell below this value for > 10-s. Verbal feedback was delivered in a standard format to remind participants to maintain an RPE of 16 at 1 min intervals throughout the trial. Participants were encouraged to constantly reassess whether they were still exercising at RPE-16. They were blinded to distance covered, elapsed time, heart rate, and power output.

### Environmental chamber

Oxygen fraction was controlled by an environmental hypoxic chamber (Sporting Edge, Basingstoke, UK). The three conditions were maintained across all trials as follows: severe hypoxia (F_I_O_2_ = 0.114; P_I_O_2_ = 82 ± 1 mmHg), moderate hypoxia (F_I_O_2_ = 0.152; P_I_O_2_ = 109 ± 1 mmHg), and ambient air (F_I_O_2_ = 0.202; P_I_O_2_ = 144 ± 2 mmHg). Temperature and humidity were controlled throughout at 19.4 ± 0.6 °C and 39 ± 4.3%, for all sessions and barometric pressure was recorded as 1013 ± 4 hPA.

### Cardiorespiratory measures

Expired gases were measured breath-by-breath to assess oxygen consumption ($\dot {V}$O_2_), minute ventilation, breathing frequency, tidal volume and end-tidal oxygen (PETO_2_), and carbon dioxide (PETCO_2_) continuously throughout the test (Vyntus CPX; CareFusion; Hochberg, Germany) and averaged into 15-s epochs across all the trials. The gas analyzer was calibrated before every trial with the gases of known concentration (15.95 O_2_, 4.97% CO_2,_ BAL. N_2_) and the turbine volume transducer was calibrated automatically by the system at flow values of 2 L s^− 1^ and 0.2 L s^− 1^. Heart rate was recorded continuously throughout the trials (Polar Heart Rate Monitor V800, Warwick, UK). SpO_2_ was sampled at 1 Hz using a finger pulse oximeter (Vyntus CPX; CareFusion; Hochberg, Germany) attached to the right index finger.

### Near-infrared spectrometry

Participants were instrumented with an NIRS optode over the right vastus lateralis to monitor the absorption of light in the muscle tissues (Portamon, Artinis Medical Systems, Zetten, The Netherlands). The optode was affixed over the muscle belly of the right vastus lateralis muscle along the vertical axis of the thigh, 2/3 between the greater trochanter and the lateral epicondyle of the femur. The optode was secured with tape and covered with an optically dense cloth to minimize the possibility that extraneous light could influence the NIRS signal. The placement position was marked with indelible ink to ensure accurate placement for future visits. The system is a two-wavelength continuous wave system that simultaneously uses the modified Beer–Lambert law and spatially resolved spectroscopy methods. Changes in tissue oxyhaemoglobin (O_2_Hb), deoxyhaemoglobin (HHb), and total haemoglobin (tHb) were measured using the differences in the absorption characteristics of infrared light at 760 and 850 nm. Differential path factor (DPF) of 4 was used throughout. NIRS data were connected to a computer by Bluetooth for acquisition at 10 Hz. Tissue oxygenation index (TSI) represents the ratio of O_2_Hb-to-tHb concentration and was reported in response to exercise and used to assume changes in intramuscular oxygen status (Ferrari et al. 2004).

### Rating of perceived exertion (RPE)

Participants were thoroughly briefed on the RPE scale before commencing the fixed-RPE trials. In line with the ACSM guidelines (American College of Sports Medicine 2000), participants were instructed to pay close attention to how difficult the exercise felt, combining total exertion, fatigue, and physical stress in hypoxia, without considering one particular factor, such as leg pain, shortness of breath, or anticipation of how they might feel several minutes later. We attempted to anchor the RPE scale by highlighting the self-reported RPE during the early stages of the incremental ramp test (RPE ~ 10–11) and the final stages of the test (RPE ~ 19–20). To further the enable visualisation of the intensity, participants were provided with associations between the RPE and intensity–duration relationships. An example of this was the guidance that an RPE of 13 was akin to a 2-h cycle, whilst holding a conversation; RPE-15 being close to a 1-h steady-state maximal effort, where sustained conversation would be difficult; and RPE-16 being a maximal effort that they could only sustain for around 25–35 min.

### Statistical analysis

All statistical analyses were performed using SPSS (IBM SPSS statistics 22 Inc, USA). A two-way analysis of variance (ANOVA) for repeated measures was used to test for within-group effects across time in and conditions. If sphericity was violated a Greenhouse–Geisser correction was applied. When a significant difference was found for a main effect (condition or time), post hoc pairwise comparisons were made, incorporating a Bonferroni adjustment. Magnitude of the effect was calculated with partial eta-squared (*η*_*p*_^2^) according to the following criteria: 0.02, small; 0.13, moderate; 0.26 large, or using Cohen’s *d* (*d*) for pairwise comparisons using: 0.2, small; 0.5, moderate; 0.8, large (Cohen 1988). Differing trial durations meant that power data were normalized with respect to time. Cardiovascular data were analyzed by averaging 30-s data at start, middle, and end points, across each trial. Correlations were performed to examine the relationship between the rate change over the first 3 min of exercise in oxygen saturation, muscle oxygenation, minute ventilation, tidal volume, breathing frequency, oxygen consumption, PETCO_2_, and heart rate to variance in exercise time in severe hypoxia, moderate hypoxia, and normoxia. Data are presented as mean ± SD (*n* = 8). Significance was set at *P* < 0.05.

## Results

Upon entering the hypoxic chamber and prior to the main experimental trial, participants conducted a short self-selected ramp to an RPE of 16 over 3–5 min. The achieved power output associated with an RPE of 16 was different between conditions (*F*_(1.180,8.262)_ = 9.558, *P* = 0.012, *η*_*p*_^*2*^ = 0.577) (Table 1), with pairwise analysis, confirming that the power output achieved in severe hypoxia was reduced relative to moderate hypoxia and normoxia. There were no differences between the peak power achieved in the ramp protocol and the peak power achieved during the experimental trial (Table 1).

**Table 1:** Peak power output during a short ramp to RPE 16 and the peak power achieved during the main experimental exercise trial

	Severe hypoxia	Moderate hypoxia	Normoxia
Peak power ramp (W)	170 ± 30*^#^	204 ± 44	207 ± 35
Peak power trial (W)	183 ± 29*^#^	201 ± 35	208 ± 25

Values are means ± SD for eight participants

*P < 0.05 relative to moderate hypoxia

^#^*P* < 0.05, relative to normoxia

### Exercise in hypoxia

**Fig. 1:**
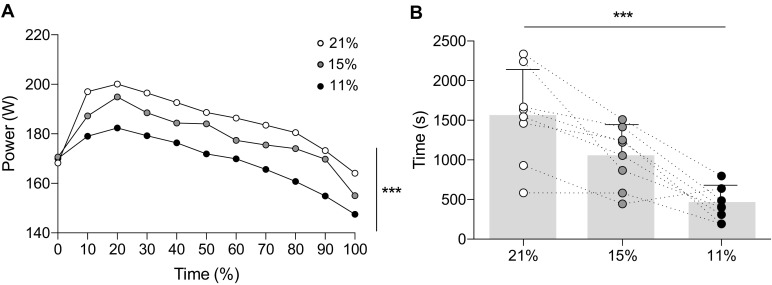
Power output during exercise at a fixed RPE in variable inspired oxygen fractions. **a** Average power output relative to exercise time expressed as 100% of the performance trial in severe hypoxia (black circle), moderate hypoxia (grey circle), and normoxia (white circle). SD has not been included for clarity. **b** Inter-individual and mean performance time across the three trials at a fixed RPE, exercise was terminated when power output dropped 20% below the peak power output achieved in the trial. All data are mean ± SD. ***Significant main effect of condition

During the exercise trial at a fixed RPE of 16, peak power output was achieved between 10 and 20% of the trial duration (Fig. 1a). Exercise time was reduced in accordance with a reduction in F_I_O_2_ (severe hypoxia = 428 ± 210 s; moderate hypoxia = 1044 ± 384 s; normoxia = 1550 ± 590 s) (*F*_(2,14)_ = 24.526, *P* < 0.001, *η*_*p*_^*2*^ = 0.778) (Fig. 1b). Modulation of power output across the exercise trial decreased with time (*F*_(10,70)_ = 32.950, *P* = 0.000, *η*_*p*_^*2*^ = 0.825) and was different between condition (*F*_(1.555,15.55)_ = 60.432, *P* = 0.000, *η*_*p*_^*2*^ = 0.858) (Fig. 1a). However, the rate of decrease in power output identified from 20% into the trial until exercise cessation (100%) was not significantly different between conditions (*P* < 0.05).

### Blood oxygen saturation

**Fig. 2:**
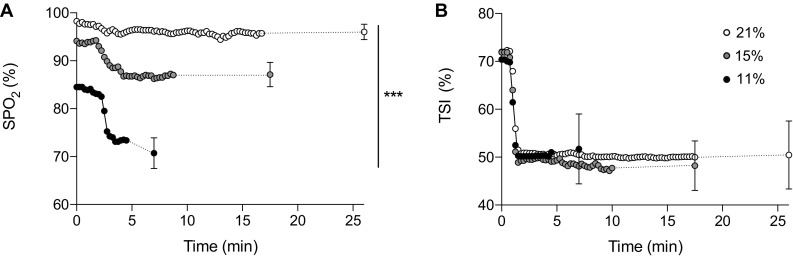
Blood oxygen saturation and vastus lateralis tissue oxygen saturation at a fixed RPE in variable inspired oxygen fractions. **a** Oxygen saturation (SpO_2_); **b** vastus lateralis tissue saturation index (TSI). Each condition is represented as severe hypoxia (black circle), moderate hypoxia (grey circle), and normoxia (white circle)

At baseline, SpO_2_ in the 30 s prior to exercise was reduced in both severe (85 ± 2.7%) and moderate hypoxia (94 ± 2.0%) relative to normoxia (98 ± 0.7%) (*F*_(2,14)_ = 132.501, *P* < 0.0001, *η*_*p*_^*2*^ = 0.950). During exercise, SpO_2_ decreased with time (*F*_(2,14)_ = 55.871, *P* < 0.0001, *η*_*p*_^*2*^ = 0.889) and decreased relative to F_I_O_2_ (*F*_(2,14)_ = 197.899, *P* < 0.0001, *η*_*p*_^*2*^ = 0.966), demonstrating an interaction effect (*F*_(4,28)_ = 18.255, *P* < 0.0001, *η*_*p*_^*2*^ = 0.723). Follow-up pairwise analysis confirmed that all conditions were different from each other (*P* < 0.05). End-exercise levels were decreased in severe hypoxia (72 ± 5%) and moderate hypoxia (87 ± 3%) relative to normoxia (96 ± 2%). The rate of oxygen desaturation the first 3 min of exercise was accelerated in severe hypoxia (− 5.3 ± 2.8% min^− 1^) relative to moderate hypoxia (− 2.5 ± 1.0% min^− 1^) with both being faster than normoxia where the change was small (− 0.7 ± 0.3% min^− 1^) (*F*_(2,14)_ = 18.571, *P* < 0.0001, *η*_*p*_^*2*^ = 0.726) (Table 1). At the point of exercise cessation relative to baseline measures, SpO_2_ was decreased by ~ 14% in severe hypoxia and ~ 8% in moderate hypoxia and maintained within ~ 3% in normoxia (Fig. 2a).

### Muscle oxygenation

Muscle oxygenation at rest was similar across all the conditions (severe hypoxia, 70 ± 3%TSI; moderate hypoxia, 72 ± 3%TSI; normoxia, 72 ± 3%TSI). TSI decreased with time (*F*_(1.122,7.855)_ = 122.771, *P* = 0.000, *η*_*p*_^*2*^ = 0.946) falling rapidly during the first few seconds of exercise in each F_I_O_2_ and reaching a steady state that was not different between each condition (*F*_(2,14)_ = 0.906, *P* = 0.426, *η*_*p*_^*2*^ = 0.115). Indeed, the rate of change in muscle oxygenation in each F_I_O_2_ was not different (*P* < 0.05). At the point of withdrawal from the task, TSI was remarkably similar between conditions (severe hypoxia, 52 ± 7%TSI; moderate hypoxia, 48 ± 5%TSI; normoxia, 51 ± 6%TSI) (Fig. 2b).

### Ventilatory measures

**Fig. 3:**
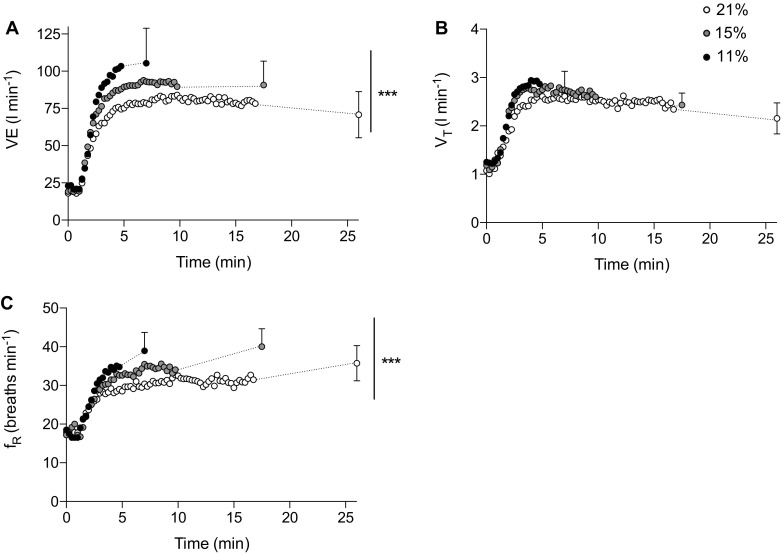
Breathing parameters at a fixed RPE in variable inspired oxygen fractions. **a** Minute ventilation, ($\dot {V}$E); **b** tidal volume (VT); **c** breathing frequency (*f*R). Each condition is represented as severe hypoxia (black circle), moderate hypoxia (grey circle), and normoxia (white circle)

**Fig. 4:**
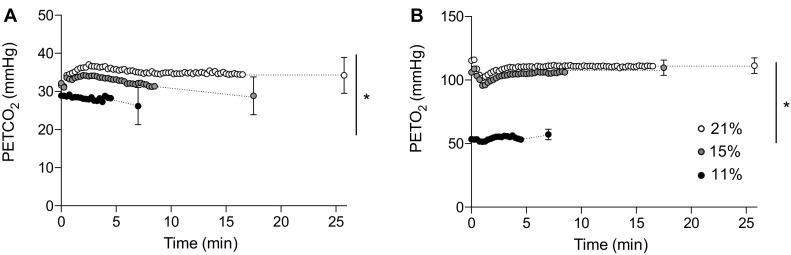
End-tidal expired air partial pressures during exercise at a fixed RPE in variable inspired oxygen fractions. **a** End-tidal carbon dioxide (PETCO_2_); **b** end-tidal oxygen (PETO_2_). Each condition is represented as severe hypoxia (black circle), moderate hypoxia (grey circle), and normoxia (white circle)

Minute ventilation increased with time (*F*_(1.107, 7.750)_ = 233.333, *P* < 0.0001, *η*_*p*_^*2*^ = 0.971) and was different between F_I_O_2_ (*F*_(2,14)_ = 25.166, *P* = 0.001, *η*_*p*_^*2*^ = 0.782) with an interaction effect (*F*_(4, 28)_ = 5.767, *P* = 0.002, *η*_*p*_^*2*^ = 0.452). Follow-up pairwise analysis confirmed that minute ventilation across the entire trial was increased in severe hypoxia (*P* = 0.002, *d* = 1.83) and moderate hypoxia (*P* < 0.001, *d* = 1.51) relative to normoxia (Fig. 3a). The rate at which minute ventilation increased over the first 3 min of exercise was accelerated relative to reducing F_I_O_2_ (*F*_(2,14)_ = 22.868, *P* < 0.001, *η*_*p*_^*2*^ = 0.766), with severe hypoxia (27.6 ± 6.6 L min^− 1^) and moderate hypoxia (21.8 ± 3.9 L min^− 1^) showing faster changes than in normoxia (17.3 ± 3.9 L min^− 1^). To understand the changes in minute ventilation, breathing frequency and tidal volume were further analyzed. Breathing frequency increased with time (*F*_(2,14)_ = 128.123, *P* < 0.0001, *η*_*p*_^*2*^ = 0.948), but was not different between condition (*P* > 0.05), but showed an interaction effect (*F*_(4,28)_ = 45.859, *P* = 0.005, *η*_*p*_^*2*^ = 0.405) (Fig. 3c). The rate of increase across the first 3 min of exercise was different in each F_I_O_2_ (*F*_(2,14)_ = 25.458, *P* < 0.001, *η*_*p*_^*2*^ = 0.784) with breathing frequency increasing by 4.4 ± 1.9 breaths min^− 1^ in severe hypoxia (*P* = 0.008, *d* = − 1.45) and 3.5 ± 1.4 breaths min^− 1^ in moderate hypoxia (*P* < 0.001, *d* = − 1.16) relative to normoxia (1.9 ± 1.5 breaths min^− 1^). Tidal volume increased with time (*F*_(2,14)_ = 12.798, *P* < 0.001, *η*_*p*_^*2*^ = 0.921) and condition (*F*_(2,14)_ = 23.586, *P* < 0.001, *η*_*p*_^*2*^ = 0.771). Follow-up pairwise analysis confirmed tidal volume was increased in severe hypoxia (*P* = 0.002, *d* = 1.65) and moderate hypoxia (*P* = 0.014. *d* = 1.03) relative to normoxia (Fig. 3b). However, the rate of change in tidal volume between condition was not different (*P* < 0.05) (Table 2). There were main effects of time for PETCO_2_ (*F*_(2,14)_ = 3.725, *P* = 0.05, *η*_*p*_^*2*^ = 0.347) and condition (*F*_(2,14)_ = 9.768, *P* = 0.002, *η*_*p*_^*2*^ = 0.583), with an interaction effect (*F*_(4, 28)_ = 4.258, *P* = 0.008, *η*_*p*_^*2*^ = 0.378). Pairwise comparisons confirmed that PETCO_2_ was reduced in severe hypoxia relative to normoxia (*P* = 0.015, *d* = − 0.98) (Fig. 4a). There was no effect of time for PETO_2_ (*F*_(2,14)_ = 1.267, *P* = 0.312, *η*_*p*_^*2*^ = 0.153), but a main effect of condition (*F*_(2,14)_ = 1210.515, *P* < 0.001, *η*_*p*_^*2*^ = 0.994), with an interaction effect (*F*_(2.014, 14.096)_ = 4.129, *P* = 0.039, *η*_*p*_^*2*^ = 0.371). Pairwise comparisons confirmed that PETO_2_ was reduced in severe hypoxia relative to normoxia (*P* < 0.001, *d* = − 17.75) and moderate hypoxia (*P* = 0.002, *d* = − 17.82) (Fig. 4b).

**Table 2:** Rate of change in physiological variables during the first 3 min of exercise in severe hypoxia, moderate hypoxia, and normoxia

	Severe hypoxia	Moderate hypoxia	Normoxia
Time (s)	428 ± 210*^#^	1044 ± 384*	1550 ± 590
Power (W% min^− 1^)	− 0.4 ± 0.1	− 0.3 ± 0.1	− 0.4 ± 0.1
SpO_2_ (% min^− 1^)	− 5.3 ± 2.8*^#^	− 2.5 ± 1.0*	− 0.7 ± 0.3
TSI (% min^− 1^)	− 3.1 ± 0.7	− 3.4 ± 1.2	− 3.6 ± 1.5
Heart rate (bpm min^− 1^)	23.6 ± 4.7	22.0 ± 6.5	22.4 ± 2.2
Minute ventilation (L min^− 1^)	27.6 ± 6.6*^#^	21.8 ± 3.9*	17.3 ± 3.9
Breathing frequency (breaths min^− 1^)	4.4 ± 1.9*^#^	3.5 ± 1.4*	1.9 ± 1.5
Tidal volume (L min^− 1^)	0.5 ± 0.1	0.6 ± 0.2	0.6 ± 0.2
$\dot {V}$O_2_ (L min^− 1^)	0.6 ± 0.1*	0.8 ± 0.1*	0.7 ± 0.1

Values are means ± SD for eight participants

$Sp{O_2}$ oxygen saturation, *TSI* tissue saturation index, $\dot {V}$*O*_*2*_ oxygen consumption

*P < 0.05 relative to normoxia; ^#^*P* < 0.05, relative to moderate hypoxia

### Cardiorespiratory measures

**Fig. 5:**
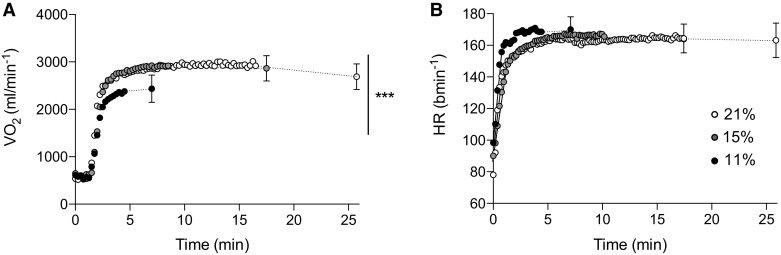
Oxygen consumption and heart rate responses during exercise at a fixed RPE in variable inspired oxygen fractions. **a** Oxygen consumption ($\dot {V}$O_2_); **b** heart rate (HR). Each condition is represented as severe hypoxia (black circle), moderate hypoxia (grey circle), and normoxia (white circle)

There were main effects of time for $\dot {V}$O_2_ (*F*_(1.073, 7.510)_ = 467.663, *P* < 0.0001, *η*_*p*_^*2*^ = 0.985) and condition (*F*_(2,14)_ = 35.680, *P* < 0.001, *η*_*p*_^*2*^ = 0.836), with an interaction effect (*F*_(4, 28)_ = 15.531, *P* = 0.002, *η*_*p*_^*2*^ = 0.689). Pairwise comparisons confirmed that $\dot {V}$O_2_ was reduced in severe hypoxia relative to both moderate hypoxia (*P* < 0.001, *d* = − 1.52) and normoxia (*P* = 0.001, *d* = − 1.32) (Fig. 5a). The rate of change in $\dot {V}$O_2_ from baseline to 3 min into exercise was different according to F_I_O_2_ (*F*_(2,14)_ = 8.800, *P* = 0.003, *η*_*p*_^*2*^ = 0.557) with a slower rate change in severe hypoxia (0.6 ± 0.1 L min^− 1^) relative to moderate hypoxia (0.8 ± 0.1 L min^− 1^) (*P* = 0.024, *d* = 1.42) and normoxia (0.7 ± 0.1 L min^− 1^) (*P* = 0.015, *d* = 1.05). $\dot {V}$CO_2_ showed main effects for time (*F*_(2,14)_ = 281.038, *P* < 0.001, *η*_*p*_^*2*^ = 0.976), but no differences between condition or rate of change (*P* > 0.05). Heart rate increased with time during exercise (*F*_(2, 14)_ = 806.597, *P* < 0.001, *η*_*p*_^*2*^ = 0.991) and plateaued at a similar point, with no differences between conditions (*P* > 0.05). Descriptively, heart rate appeared to increase and reach steady state faster in severe hypoxia (Fig. 5b); however, there was no significant change in rate during the first 3 min of exercise between conditions (*P* > 0.05).

### Rate change effects on exercise performance

Moderate-to-strong correlations were identifed between the rate of change during the first 3 min of exercise in breathing frequency (*r* = − 0.718, *P* < 0.001) and S_p_O_2_ (*r* = 0.611, *P* = 0.002) and exercise duration at a fixed RPE in differing F_I_O_2_.

## Discussion

The purpose of the study was to investigate the effect of severe and moderate hypoxia on exercise performed at a fixed RPE in reference to normoxia. As anticipated, our findings demonstrate that performance time was diminished when exposed to decreasing F_I_O_2_, meaning that participants down-regulated their work load as a result of increasing levels of hypoxia. Increases in breathing frequency and blood oxygen desaturation during the early stages of exercise were correlated with reductions in task performance. Despite these changes, oxygen extraction at the muscle (as indicated by NIRS) appeared to be tightly regulated to match the metabolic demand, suggesting that muscle oxygenation is not involved in determining perception during the early stages of setting exercise intensity. Together, the early rate of change in ventilation and arterial hypoxemia appears to drive the selection of exercise intensity associated with a fixed RPE in hypoxia.

Reductions in exercise performance in hypoxia have been attributed to depleted arterial oxygen content (Fulco et al. 1996, 1998; Calbet et al. 2003a; Amann et al. 2006b; Romer et al. 2006). Breathing hypoxic gas leads to a decrease in the arterial partial pressure of oxygen, oxygen saturation of haemoglobin, and the amount of oxygen dissolved in the plasma. Consequently, arterial oxygen content is reduced. Here, a reduction in F_I_O_2_ decreased exercise time by ~ 72% in severe hypoxia (F_I_O_2_ < 0.115) and by ~ 33% in moderate hypoxia (F_I_O_2_ ~ 0.15), relative to normoxia. There was some evidence of inter-individual responses with two participants showing a reduced sensitivity to hypoxia. Whilst a number factors have been presented to explain responders and non-responders to hypoxia and altitude (Fulco et al. 1998), these individuals performed relatively poorly in normoxia and recorded low aerobic capacity, suggesting that this may be related to fitness status. Across the group, SpO_2_ was maintained within 3% of resting levels during normoxia; however, upon acute exposure to moderate hypoxia, resting S_P_O_2_ was reduced by ~ 4% and decreased by a further 8% across the exercise trial. In severe hypoxia, these reductions in SpO_2_ were much greater, decreasing by ~ 14% at rest, with a further decrease of 14% (S_P_O_2_ ~ 72%) observed at end-exercise. Reductions in exercise performance in moderate hypoxia have largely been attributed to peripheral mechanisms, where a decrease in arterial oxygen content and impaired oxygen delivery to the working muscle leads to a subsequent metabolic perturbation (Hogan et al. 1999). This work has been advanced by studies describing comparable levels of peripheral muscle fatigue via evoked maximal contractions following exhaustive exercise in normoxia and hypoxia despite a substantial reduction in exercise time (Amann et al. 2006b; Romer et al. 2006, 2007; Goodall et al. 2012). Greater reductions in performance described in severe hypoxia have been attributed to greater impairments in pulmonary gas exchange, reduced limb blood flow, and reductions in cardiac output (Calbet et al. 2003a). However, experiments demonstrate rapid improvements in exercise performance and cerebral oxygenation following a fast transition from breathing gas that is severely hypoxic to hyperoxic, at the point of exhaustion, supporting a central role (Amann et al. 2006a).

We examined how the initial exposure to a hypoxic environment would impact determination of the exercise intensity associated with 16 on the Borg RPE scale (Borg 1982). Changes in perceived exertion may be determined during the beginning stages of exercise via a range of perceptual, peripheral, experiential, and environmental sensory cues to enable task completion within the physiological limits of the body (Hampson et al. 2001; St Clair Gibson et al. 2006). In both normoxic and moderate hypoxic conditions, the selected power output achieved within several minutes was comparable; however, in severe hypoxia, power output was reduced by ~ 18%. Whilst the ability to generate maximal power is unaffected by severe levels of hypoxia (Calbet et al. 2003b), supporting the argument that motor drive is unaffected upon acute exposure, the heightened perception of effort observed here may reflect a detrimental effect on decision-making processes (Niedermeier et al. 2017), cognition (McMorris et al. 2017), or a teleoanticipatory reduction in power output to maintain homeostasis (St Clair Gibson et al. 2006). During exercise in hypoxia, cerebral vascular conductance is continually adjusted to maintain oxygen delivery when a reduction in arterial oxygen concentration occurs (Curtelin et al. 2018). However, reductions in cerebral oxygenation have been described at rest and during exercise in hypoxia (Subudhi et al. 2009). Whilst the brain can compensate by increasing oxygen extraction (Gonzalez-Alonso et al. 2004), neuronal function can also be inhibited (Neubauer et al. 1990) which may impact higher cognitive functions. Whilst we did not directly measure cerebral oxygenation or cerebral blood flow, we did observe a reduction end-tidal carbon dioxide (PETCO_2_) in severe hypoxia relative to normoxia. A close relationship exists between PETCO_2_ and cerebral blood flow (Ide et al. 2003), suggesting that cerebral blood flow may have been reduced during exercise in severe hypoxia. Hypocapnia reduces cerebral blood flow by as much as ~ 3% for every ~ 1 mmHg change in PETCO_2_ (Ringelstein et al. 1992). Based on an observed ~ 8 mmHg difference during exercise in normoxia and severe hypoxia (Fig. 4a), this could equate to a ~ 24% reduction in cerebral blood flow. It should also be noted that PETCO_2_ sensitivity is increased by acute exposure to hypoxia (Jensen et al. 1996; Poulin et al. 2002) and the effect on PETCO_2_ sensitivity has shown differential effects (Fortune et al. 1992; Vovk et al. 2002). Therefore, these observations should be further explored.

At submaximal exercise intensities when arterial oxygenation is reduced, it is probable that oxygen delivery to exercising muscles is compromised. However, compensatory mechanisms increase oxygen extraction or blood flow to maintain muscle oxygen supply (Amann and Calbet 2008). Remarkably, vastus lateralis oxygen saturation assessed via NIRS, did not differ, irrespective of the level of arterial hypoxemia experienced, suggesting comparable oxygenation in the primary exercising muscles during cycling. This has been previously observed during submaximal exercise in both severe and moderate hypoxia (Millet et al. 2012). Here, it was reasoned that afferent signalling, emanating from the metabolic environment at the muscle, is unlikely to have changed between conditions and, therefore, could not explain the reductions in exercise performance. On this basis, it was postulated that tissue deoxygenation was unlikely to play a role in determining exercise intensity. However, the metabolic adaptations and changes in blood flow to maintain constant tissue oxygenation may have contributed to afferent signalling. In the current study, the rate of change in TSI% during the first 3 min of exercise did not differ according to F_I_O_2_, yet it transitioned to a lower steady state from the onset of exercise across all the conditions. Therefore, working muscles were able to match oxygen delivery and extraction to meet the metabolic demand during submaximal exercise, despite an increasing physiological perturbation.

Reductions in F_I_O_2_ also corresponded with increases in minute ventilation. End-exercise minute ventilation was augmented by 41% and 24% in severe and moderate hypoxia, respectively, compared to normoxia. Ventilation during exercise is controlled via a balance of centrally mediated feed-forward commands and peripheral feedback that increases the rate and depth of breathing according to the demands imposed by the exercise intensity (Kaufman and Forster 1996). Changes in the partial pressure of blood gases are sensed by both central and peripheral chemoreceptors. In the brain, chemoreceptors respond to changes in brain tissue CO_2_/[H^+^] (Nattie and Li 2009; Tipton et al. 2017). Peripherally, chemoreceptors located in the carotid artery respond to low arterial O_2_ and high arterial CO_2_. The increased ventilatory response during the majority of the exercise trial was achieved by an increased tidal volume, which is typically the most efficient mechanical way to increase minute ventilation commensurate with metabolic needs and decreasing arterial blood gas tensions (Tipton et al. 2017). During the early stages of exercise, the rate of increase in breathing frequency was inversely related to the reductions in ambient F_I_O_2_ and was the strongest predictor of exercise time in hypoxia. The sensations of ventilation and breathing discomfort are consciously monitored during exercise (Robertson 1982). Indeed, the relationship between RPE and ventilation is well established (Cafarelli and Noble 1976; Robertson 1982; Killian 1998; Nicolo et al. 2016). In hypoxia, it is, therefore, possible that the rapid change in ventilation during exercise may have potentiated a greater conscious awareness, contributing to both the initial setting of exercise intensity and the modulation of perceived exercise intensity thereafter.

In an attempt to further examine the acute effects of hypoxia, we determined the rate change of the physiological measures taken during the first 3 min of exercise when an approximate steady state was obtained. We found that rate changes in breathing frequency and blood oxygen desaturation showed moderate-to-strong correlations with performance time. A recent study by Farra et al. (2017) elegantly demonstrated that, when the rate of SpO_2_ was altered via F_I_O_2_, faster arterial deoxygenation resulted in a greater decline in perceptually controlled exercise performance (Farra et al. 2017). They suggested that RPE was sensitive to both the rate of change and absolute magnitude of arterial deoxygenation, which we can partially support based on a fixed hypoxic environment. Interestingly, in contrast to our findings, they reported no difference in breathing frequency between the experimental conditions (fast, medium and slow desaturations). This may reflect the gradual reduction in S_P_O_2_ controlled by Farra et al. (2017), which may have masked the relative contributions of the other candidate sensory cues, such as breathing rate, that combine to inform higher brain centres of the homeostatic disturbances. Indeed, these early cues appear to influence the selection of the initial exercise intensities and the subsequent power output that is sustainable for the entire exercise trial. Future work should explore the relationship between breathing frequency and exercise time in hypoxia and practical solutions to reduce breathing frequency may facilitate improvements to performance.

The relationship between exercise regulation, pacing, and RPE is still heavily debated. A three-dimensional framework has recently been proposed as a multidimensional model of volitional self-regulatory control and perceived fatigability (Venhorst et al. 2018). The model combines a sensory-discriminatory dimension (peripheral and central sensations), an affective-motivational dimension (arousal and motivation), and a cognitive-evaluative dimension (exertion and task aversion) (Venhorst et al. 2018). Importantly, this model accounts for both external and internal mediating factors in the generation of RPE. These multiple inputs are, therefore, continually processed, integrated, and interpreted, consciously or otherwise, to alter pacing behaviour in anticipation of potential threats to homeostasis (Hampson et al. 2001; Noakes 2004; Tucker 2009; Venhorst et al. 2018). Such complex psychophysiological interactions, therefore, provide a construct for the observed behavioural differences in pacing in severe and moderate hypoxia when exercising according to a fixed RPE. As we reported, the rate of decrease in power output did not differ between conditions once peak power was achieved; hence, the early setting of an acceptable perceived exercise intensity appears crucial to exercise performance. Whilst it is likely that the exercising template is updated as exercise ensues (Brick et al. 2016), the interplay between such dimensions in generating RPE when challenged with reduced F_I_O_2_ will require further investigation. Therefore, we propose that the early setting of task intensity in a hypoxic environment is chiefly based upon two primary physiological cues of ventilation and SpO_2,_ thus determining performance.

## Limitations

Attenuated perceptual responses after hypoxic training have been described (Brocherie et al. 2017), suggesting an improved tolerance or acclimation to hypoxia after only one session. The subsequent effects of prior exposure on perception and exercise intensity using the fixed-RPE protocol are unknown and warrant further investigation. Whilst the measures of blood oxygen saturation and muscle tissue oxygenation were recorded in this study, we did not measure cerebral oxygenation. Our speculation regarding the greater reduction in exercise during exposure to severe hypoxia is largely supported by a theoretical reduction in brain oxygenation. In comparable levels of hypoxia, others (Amann et al. 2007; Subudhi et al. 2007, 2009; Goodall et al. 2012) have reported such changes, therefore, despite this being a limitation to our findings, it is plausible that comparable levels of cerebral oxygenation may have occurred. We also were able to examine PETCO_2_ data which closely align with change in cerebral blood flow as previously discussed. In addition, whilst we noted the comparable levels of muscle tissue oxygenation during exercise in hypoxia, further analysis will be required to determine if a stable oxygen condition was met by increases in blood flow or reduced metabolic demand, which may give further insight into the afferent processes that may underlie this observation. Finally, measures of peripheral muscle fatigue would have been useful to quantify the level of fatigue experienced and further support our conclusions regarding peripheral and central mechanisms.

## Conclusions

In conclusion, severe and moderate hypoxia elicited reductions in exercise when controlled at a fixed RPE. The primary cues for determining perceived effort related to the decrease in arterial hypoxemia and increase in ventilation, which was largely driven by an increase in breathing frequency. The strong relationships found between exercise time and both ventilation and SpO_2_ support the role of these physiological cues in setting the early intensity of perceptually controlled exercise.

## Abbreviations

ANOVA: Analysis of variance
CNS: Central nervous system
*d*: Cohen’s *d*
${{\text{F}}_{\text{I}}}{{\text{O}}_{\text{2}}}$: Fraction of inspired oxygen
$${{\text{H}}^+}$$: Hydrogen
HHb: Deoxyhaemoglobin
NIRS: Near-infrared spectroscopy
${{\text{O}}_{\text{2}}}{\text{Hb}}$: Arterial oxygen saturation
${\eta _p}^{{\text{2}}}$: Partial eta-squared
PETCO_2_: End-tidal carbon dioxide
PETO_2_: End-tidal oxygen
PNS: Peripheral nervous system
RPE: Rating of perceived exertion
$${\text{Sp}}{{\text{O}}_{\text{2}}}$$: Oxygen saturation
tHb: Total haemoglobin
TSI: Tissue saturation index
$$\dot {V}{{\text{O}}_{\text{2}}}$$: Oxygen consumption
$\dot {V}{{\text{O}}_{{\text{2max}}}}$: Maximal oxygen uptake
$\dot {V}{\text{C}}{{\text{O}}_{\text{2}}}$: Expired carbon dioxide
